# Plio-Pleistocene sea level and temperature fluctuations in the northwestern Pacific promoted speciation in the globally-distributed flathead mullet *Mugil cephalus*

**DOI:** 10.1186/1471-2148-11-83

**Published:** 2011-03-31

**Authors:** Kang-Ning Shen, Brian Wade Jamandre, Chih-Chieh Hsu, Wann-Nian Tzeng, Jean-Dominique Durand

**Affiliations:** 1 Institute of Fisheries Science, College of Life Science, National Taiwan University, Taipei 106, Taiwan, China; 2 Institut de Recherche pour le Développement (IRD), UMR 5119 ECOSYM, Campus IRD/ISRA de Bel Air, BP 1386, CP 18524 Dakar, Sénégal

## Abstract

**Background:**

The study of speciation in the marine realm is challenging because of the apparent absence of physical barriers to dispersal, which are one of the main drivers of genetic diversity. Although phylogeographic studies using mitochondrial DNA (mtDNA) information often reveal significant genetic heterogeneity within marine species, the evolutionary significance of such diversity is difficult to interpret with these markers. In the northwestern (NW) Pacific, several studies have emphasised the potential importance of sea-level regression during the most recent glaciations as a driver of genetic diversity in marine species. These studies have failed, however, to determine whether the period of isolation was long enough for divergence to attain speciation. Among these marine species, the cosmopolitan estuarine-dependent fish *Mugil cephalus *represents an interesting case study. Several divergent allopatric mtDNA lineages have been described in this species worldwide, and three occur in sympatry in the NW Pacific.

**Results:**

Ten nuclear microsatellites were surveyed to estimate the level of genetic isolation of these lineages and determine the role of sea-level fluctuation in the evolution of NW Pacific *M. cephalus*. Three cryptic species of *M. cephalus *were identified within this region (NWP1, 2 and 3) using an assignment test on the microsatellite data. Each species corresponds with one of the three mtDNA lineages in the COI phylogenetic tree. NWP3 is the most divergent species, with a distribution range that suggests tropical affinities, while NWP1, with a northward distribution from Taiwan to Russia, is a temperate species. NWP2 is distributed along the warm Kuroshio Current. The divergence of NWP1 from NWP2 dates back to the Pleistocene epoch and probably corresponds to the separation of the Japan and China Seas when sea levels dropped. Despite their subsequent range expansion since this period of glaciation, no gene flow was observed among these three lineages, indicating that speciation has been achieved.

**Conclusions:**

This study successfully identified three cryptic species in *M. cephalus *inhabiting the NW Pacific, using a combination of microsatellites and mitochondrial genetic markers. The current genetic architecture of the *M. cephalus *species complex in the NW Pacific is the result of a complex interaction of contemporary processes and historical events. Sea level and temperature fluctuations during Plio-Pleistocene epochs probably played a major role in creating the marine species diversity of the NW Pacific that is found today.

## Background

In the marine environment, fluctuations in sea level, water temperatures and sea ice caused by glacial cycles are believed to have had a major influence on species distributions and the population connectivity of marine species [1-3]. For instance, lowered sea levels during glacial maxima led to the emergence of land bridges, which fragmented marine ecosystems and isolated populations of aquatic species [4-6]. At the same time, temperature fluctuations in the Pleistocene epoch influenced population dynamics by promoting bottlenecks and the loss of genetic heterogeneity. In fact, much of the intraspecific genetic diversity that is exhibited by contemporary species is generally assumed to have derived from such events in the Pleistocene (for a review, see [7]).

**Figure 1:**
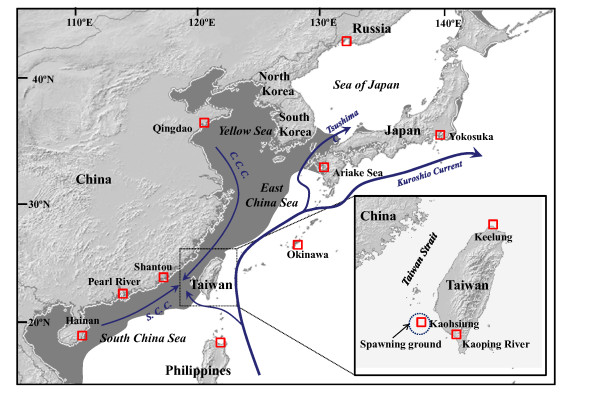
**Sampling localitions of *Mugil cephalus *in the northwestern Pacific**. The shaded zone in dark grey is the area of the continental shelves that was exposed during periods of low sea levels. Blue arrows correspond to currents present in the area, C. C. C.: China Coastal Current, S. C. C.: South China Current.

The northwestern (NW) Pacific is characterised by marginal seas, which were particularly impacted by Plio-Pleistocene glacial cycles. The lowering of sea level caused the recurrent closure of the Japan Sea, the semi-closure of the South China Sea and the partial or full exposure of the East China and Yellow Seas [8]. The closure of the Japan Sea caused fluctuations in sea temperatures, by halting the influx of the warm Tsushima Current, a branch of the Kuroshio Current (Figure 1) that supplies a large amount of heat to northern areas [9]. As a consequence, seascape dynamics of the region are believed to have profoundly influenced both intraspecific genetic diversity and species diversity of marine organisms, as exemplified by the exceptional diversity found in SE Asia [10-12]. This hypothesis has been confirmed by recent phylogeographic investigations in the NW Pacific area (Table 1). The significance of the lowering of the sea level and glaciations for species diversity has generally, however, not been assessed. The overlapping distribution of multiple divergent lineages is a striking phylogeographic pattern that is common to most of the organisms that have been investigated in this region, and it raises the question of whether some of these lineages are in fact cryptic species. For example, some authors [13-15] considered the various divergent lineages to be different species of Pleistocene origin, while others [16,17] have interpreted these lineages as intraspecific polymorphisms, consistent with the existence of isolated populations during glacial maxima and secondary contact at the end of the Pleistocene. Finally, the comparison of phylogeographic information among different marine species inhabiting the same area can provide for conflicting interpretations of the role of decreasing sea level and Pleistocene glaciations, and highlights the difficulty of distinguishing species in the early stages of differentiation from populations experiencing secondary contact after an extended isolation.

**Table 1:** List of phylogeographic studies that investigated the genetic diversity of NW Pacific marine species and summary of the relevant information reported in each study.

Species	Organism	**Genetic info**.	N	Structure/Refugium	NL	**% div**.	**cryptic sp**.	References
*Mugil cephalus*	Fish	control region	126	East China Sea vs South China Sea	2	48%	yes	[14]
*Mugil cephalus*	Fish	control region	140	East China Sea vs South China Sea	2	55%	no	[21]
*Mugil cephalus*	Fish	cyto *b*	98	Japan Sea vs East China Sea vs South China Sea	3	5.1-6.6%	no	[17]
*Mugil cephalus*	Fish	COI	448	Japan Sea vs South China Sea vs SW Pacific origin	3	2.5-4.8%	yes	This study
*Mugil cephalus*	Fish	16 S, 12 S	9	East China Sea vs South China Sea	2	1.87%, 1.61%	yes	[71]
*Mugil cephalus*	Fish	microsatellites	713	Japan Sea vs South China Sea vs SW Pacific origin	3	11.5-30.5%	yes	This study
*Mugil cephalus*	Fish	AFLP	118	Bohai Sea vs Yellow Sea vs East China Sea vs South China Sea	4	-	no	[23]
*Chelon haematocheilus*	Fish	control region	272	Japan Sea vs East China Sea vs South China Sea	3	1.55-2.41%	no	[16]
*Chelon haematocheilus*	Fish	16 S, 12 S	8	East China Sea vs South China Sea	2	2.81%, 4.11%	yes	[71]
*Odontamblyopus sp*.	Fish	ND5	85	East China Sea vs South China Sea	3	2.37-8.96%	yes	[15]
*Lateolabrax sp*.	Fish	control region	256	Japan Sea vs East China Sea	2	22.60%	yes	[72]
*Lateolabrax sp*.	Fish	cyto b	256	Japan Sea vs East China Sea	2	7.80%	yes	[72]
								
*Eriocheir sensu stricto*	Crustacean	COII, cyto *b*	154	Japan Sea vs East China Sea vs South China Sea	3	3.2-4.05%	yes	[13]
*Eriocheir sensu stricto*	Crustacean	COI, cyto *b*	446	Japan Sea vs East China Sea vs South China Sea vs Ryukyu	4	-	no	[54]
*Penaeus japonicus*	Crustacean	control region	95	Japan Sea vs East China Sea vs South China Sea	3	*F*_CT _= 0.04	no	[73]
								
*Cyclina sinensis*	Mollusk	RAPD	50	Yellow Sea vs South China Sea	2	10.90%	no	[74]
*Cyclina sinensis*	Mollusk	AFLP	160	Yellow Sea vs China Sea	2	-	no	[75]
*Tegillarca granosa*	Mollusk	COI	38	East China Sea vs South China Sea	2	15%	no	[76]
*Tegillarca granosa*	Mollusk	RAPD	96	East China Sea vs South China Sea	2	32.6 -45.1%	no	[77]
*Helice sp*.	Mollusk	COI, 16 S, 12 S, ITS2	123	East China Sea vs South China Sea vs Ryukyu & Taiwan Islands	3	0.16-1.13%	yes	[78]

N: sample size, NL: number of lineage, % div.: percentage of divergence.

The flathead mullet, *Mugil cephalus *Linnaeus, 1758, is a euryhaline teleost distributed worldwide in coastal waters, lagoons, bays and estuaries between latitudes 42° N and 42° S [18]. It is subjected to intense and unregulated inshore fisheries in the NW Pacific because of the economic value of its roe. There have been drastic declines in the landings of *M. cephalus *since 1980 (6 865 metric tons in 1980 versus 159 metric tons in 2008; Taiwan Fisheries Agency), which has been suggested to be the result of overfishing and the impact of global warming [19,20]. Thus, a sound management program is urgently needed, which requires precise knowledge about the taxonomy and population structure of this over-exploited species.

Previous studies have shown that up to three highly divergent mitochondrial lineages exist in sympatry in NW Pacific *M. cephalus *populations [14,17,21]. This high inter-lineage divergence raises questions about the taxonomic status of *M. cephalus *in the NW Pacific, but studies have not reached consensus on this issue. The inter-lineage divergence of 48% observed at the mtDNA control region probably indicates a species complex, because such divergence coupled with the existence of different reproductive behaviours (resident versus migratory) demonstrates that these lineages are independent [14]. By contrast, an inter-lineage divergence of 5.3 to 6.7% was estimated using cytochrome *b *sequences, which would argue for the existence of a single species because the genetic divergence between mugilid species exceeds this by a factor of two [17]. However, because the mtDNA genome used in both studies is maternally inherited, it is not possible to determine whether the presence of divergent mitochondrial lineages in the same sample is a result of secondary contact after an extended period of isolation and/or the presence of two sibling species.

To our knowledge, only two studies on *M. cephalus *in this region have used biparentally inherited markers [22,23]. The first study used an allozyme locus, GPI-A (glucose-6-phosphate isomerase), which may be able to distinguish migratory from resident *M. cephalus *populations [22]. More recently, Liu et al. [23] found a high level of genetic structure in *M. cephalus *using AFLP, and four populations were identified among a set of 6 samples collected along the Chinese coast, with the southern samples (Hainan Island) being the most divergent. This marked genetic structuring in NW Pacific *M. cephalus *contrasts with findings from the Gulf of Mexico and the northwest Atlantic, which did not reveal any genetic heterogeneity over a similar geographic scale, and found only one mitochondrial lineage [24,25].

Therefore, given all of the above uncertainties, this study investigated the level of both historical and contemporary gene flow among *M. cephalus *samples collected in the NW Pacific, using a set of ten microsatellites and one mtDNA (COI) locus. The objective was to estimate the level of independence of the different *M. cephalus *mtDNA lineages, and reveal the existence of cryptic species. This provided an opportunity to address both the evolutionary forces and reproductive boundary uncertainties among and within the mitochondrial lineages. Finally, we aimed to better understand the importance of Pliocene and Pleistocene geologic and climatic events in the diversification of marine species in the NW Pacific.

## Methods

### Sampling and DNA extraction

A total of 713 sub-adult to adult *M. cephalus *were collected from 12 locations in the NW Pacific (Table 2, Figure 1). Additional temporal sampling (2005-2008) was conducted at three localities around Taiwan: the coast of Keelung (KL), which is located midway along the *M. cephalus *spawning migration route from the Eastern China Sea; the offshore waters of Kaohsiung (KS), where spawning occurs; and the Kaoping Estuary (KP), a tropical system located in southwestern Taiwan. Sampling locations, dates of collection, sample sizes for microsatellites analyses, mtDNA lineage (highlighted either by sequencing or multiplex COI haplotype-specific PCR), mean fork length (mm) and gonadosomatic index (GSI) data for the *M. cephalus *from Taiwan are shown in Table 2. Muscle tissues were preserved in 95% ethanol prior to DNA extraction. Genomic DNA was extracted from muscle tissue using a DNA Purification Kit (Bioman, Taipei, Taiwan), preserved in TE buffer and then quantified and diluted to 1 ng/μl for PCR.

**Table 2:** Summary of the collected data and sample sizes of *Mugil cephalus *used in microsatellite (MS) and mitochondrial (mtDNA) COI gene analyses.

Country	code	Locations/Habitat	Sampling date	**Long. - Lat**.	**N indiv**.		% mtDNA Lineage			Fork length	GSI	GSI mean ± se/		
					microsat	COI/RS	1	2	3	mean ± se (mm)	mean ± se	mtDNA 1	mtDNA 2	mtDNA 3
Taiwan	05 KL	Keelung/	Dec. 2005	25°11'N 121°47'E	50	50/50	92	8	0	464.80 ± 37.89	15.67 ± 5.97	15.91 ± 5.82	12.86 ± 7.94	-
	06 KL	Coastal waters	Dec. 2006		48	11/48	98	2	0	478.77 ± 32.81	15.16 ± 7.80	15.32 ± 7.81	7.49	-
	07 KL		Dec. 2007		47	47/47	77	19	4	484.6 ± 68.37	10.60 ± 6.45	12.31 ± 5.55	5.87 ± 6.58	1.03 ± 0.27
	
	05 KS	Kaohsiung/	Dec. 2005	22°37'N 119°30'E	44	36/44	100	0	0	475.73 ± 34.55	20.61 ± 0.68	20.61 ± 0.68	-	-
	06 KS	Offshore waters	Dec. 2006		50	20/50	100	0	0	489.25 ± 25.37	19.75 ± 4.23	19.75 ± 4.23	-	-
	07 KS		Dec. 2007		57	49/57	100	0	0	526.01 ± 38.07	18.03 ± 1.44	18.03 ± 1.44	-	-
	
	05 KP	Kaoping River/	Dec. 2005	22°28'N 120°25'E	45	36/45	0	49	51	289.34 ± 19.58	0.81 ± 1.97	-	1.01 ± 1.52	0.73 ± 2.37
	07 KP	Estuary	Dec. 2007		49	39/49	14	63	23	501.07 ± 55.12	8.30 ± 7.50	7.31 ± 5.94	6.73 ± 6.19	13.37 ± 7.65
	08 KP		Jan. 2008		38	37/38	13	71	16	481.69 ± 39.00	0.01 ± 0.01	0.02 ± 0.03	0	0.01 ± 0.01

Japan	OK	Okinawa	Nov. 2005	26°24'N 127°54'E	48	13/48	27	73	0	-	-	-	-	-
	YK	Yokosuka	Jan.-Feb. 2005	36°16'N 139°41'E	43	8/43	35	65	0					
	AS	Ariake Sea	Nov. 2007	33°00'N 130°26'E	21	19/21	100	0	0	-	-	-	-	-

China	HN	Hainan	Apr. 2010	18°65'N 110°37'E	32	32/32	0	41	59	-	-	-	-	-
	PR	Pearl River	Feb. 2005	22°45'N 113°37'E	30	16/30	0	90	10					
	ST	Shantou	Jan. 2008	23°20'N 116°44'E	48	7/48	0	91	9	-	-	-	-	-
	QD	Qingdao	Feb. 2006	36°02'N 120°21'E	32	10/31	100	0	0	-	-	-	-	-

Philippines	PH	Philippines	Feb. 2007	18°21'N 121°37'E	14	14/14	0	100	0	-	-	-	-	-

Russia	RU	Russia	Jun. 2007	42°53'N 132°44'E	4	4/4	100	0	0	-	-	-	-	-
			Jul.-Aug. 2009		13	0/13	100	0	0	-	-	-	-	-

Total					713	448/712								

RS: rapid PCR screening of different lineages. The ratio of different lineages in each sample, their fork length and Gonadosomatic index (GSI) are also shown.

### Mitochondrial DNA analysis

Partial mtDNA COI gene sequences (627 bp) were amplified by polymerase chain reaction (PCR) using the universal primers FishF1 and FishR1 [26]. To identify *M. cephalus *cytochrome *b *lineages [17], cytochrome *b *gene was also sequenced for 20 individuals belonging to three COI lineages using the cytochrome *b *primers MCglu-1.F: GGCTTGAAAAACCACCGTTG and MCcytbR: AGTACTGTGGCAGAGCTTGG. PCR was performed in a Biometra TGradient Thermocycler with a 15-μL reaction volume containing 0.2 μm dNTPs, 1.5 μL of 10× PCR buffer (Bioman, Taipei, Taiwan), 0.5 μm each of forward and reverse primer, 0.2 U Taq DNA polymerase (Bioman, Taipei, Taiwan), and 1.0 μL of template DNA. MtDNA amplification was carried out using the following PCR conditions: 35 cycles of denaturation at 94°C for 15 s, annealing at 58°C for 15 s and extension at 72°C for 30 s after heating at 94°C for 5 min. The PCR products were electrophoresed on a 1.0% agarose gel (Bioman, Taipei, Taiwan) and stained with ethidium bromide for band characterisation via ultraviolet trans-illumination. All sequencing reactions were performed according to the manufacturer's protocol (Applied Biosystems, Foster City, CA, USA) using the same forward primer as used for PCR.

All COI sequences were automatically aligned using MAFFT version 6 [27] and manually corrected. The characterisation of the genetic variability of the COI gene sequences and the number of nucleotide substitutions, including transition/transversions, were conducted using ARLEQUIN version 3.01 [28]. Phylogenetic trees were reconstructed using the Bayesian, neighbour joining (NJ), maximum likelihood (ML) and maximum parsimony (MP) methods. The Bayesian method was applied using the program MrBayes 3.0 [29], and NJ, ML and MP were applied using the program PAUP 4.0 [30]. The transversion model with invariable sites HKY + I + G (I = 0.097, G = 0.244, Ts/Tv ratio = 5.4465) was selected for construction of the phylogenetic tree using *Mugil curema *as an outgroup (GenBank accession numbers: EU715464, [31]) and the Akaike information criterion (AIC) in MODELTEST 3.7 [32]. Nodes with bootstrap values ≥ 70% were considered well supported [33].

The fixation index (Φ_ST_) for all pair-wise comparisons among different populations was calculated to investigate genetic diversity among *M. cephalus *populations, and a permutation test (10 000 permutations) was performed using ARLEQUIN version 3.01 [28]. Population genetic diversity was measured within each of the populations based on the number of distinct haplotypes, gene diversity (*h*) and mean nucleotide diversity (π), using ARLEQUIN version 3.01 [28].

The divergence time for the *M. cephalus *COI gene was estimated using Bayesian Evolutionary Analysis Sampling Trees (BEAST) ver. 1.5.2 [34] with 20 million steps in a Monte Carlo Markov Chain (MCMC) simulation (2 million step burn-in time). COI sequences of *M. cephalus *samples from disjunct locations in the western Atlantic (South Carolina: GenBank accession numbers: HQ149710 and Florida: GenBank accession numbers: HQ149711) and eastern Pacific (Peru: GenBank accession numbers: HQ149714 and Mexico: GenBank accession numbers: HQ149715) were used for the calibration, and assuming that the divergence of these populations corresponds to the closure of the Isthmus of Panama, 2.8 MY ago [35]. A strict molecular clock was assumed for this final run. The effective sample sizes (ESS) of parameters sampled from the MCMC were > 500 (acceptable ESS is >200). The results were viewed using TRACER 1.4 [36]. Historical demographic/spatial expansions of *M. cephalus *were explored using two different approaches. Tajima's *D *[37] and Fu's *F*_S _[38] tests were used to test population equilibrium. Deviations from the sudden population expansion model were tested using the Harpending's raggedness index RI of mismatch distributions [39].

### Rapid screening of the different mtDNA lineages

A multiplex COI haplotype-specific PCR (MHS-PCR) was designed following the recommendations of previous studies [40,41] to develop a rapid screening method capable of detecting different mitochondrial lineages of *M. cephalus*. Specific mutations of the different *M. cephalus *mtDNA lineages were identified using 448 COI sequences of *M. cephalus *produced in this study. NWP1,2F (5' GCTTTTCCCCGAATAAAT 3') was the forward primer for both lineage 1 and lineage 2, while NWP3F (5'TACTGCCCTAAGCCTACTC 3') was the forward primer for lineage 3. NWP1,3R (5' CGATCTGTTAGGAGTATGG 3') was the reverse primer for lineage 1 and lineage 3, while NWP2R (5' CTCATACGAAAAGGGGTGTT 3') was the reverse primer for lineage 2. PCR was performed using a Biometra TGradient Thermocycler with a 15-μL reaction volume containing 0.2 μm of each dNTP, 1.5 μL 10× PCR buffer (Bioman, Taipei, Taiwan), 0.5 μm forward and reverse primers, 0.2 U Taq DNA polymerase (Bioman, Taipei, Taiwan), and 1.0 μL of template DNA. MtDNA amplification was carried out using the following PCR program: 35 cycles of denaturation at 94°C for 15 s, annealing at 55°C for 15 s and extension at 72°C for 30 s after heating at 94°C for 5 min. The PCR products were electrophoresed in a 1.0% agarose gel (Bioman, Taipei, Taiwan) and stained with ethidium bromide. Lineage 1 had a PCR product of 362 bp, while lineage 2 and lineage 3 had PCR products of 283 bp and 549 bp, respectively (Additional file 1, Figure S1). The rest of the samples that were not sequenced were all rapidly screened to determine their lineages.

### Microsatellite analysis

Ten microsatellite loci were used to screen the genetic diversity of the *M. cephalus *samples [42]. Reverse primers for each locus were labelled with fluorescent dyes (6-FAM, HEX and TAMRA), and multiplex PCR was performed in a 15-μl reaction volume containing 0.1 ng DNA, 1.25 pmole each of the three reverse primers labelled with different fluorescent dyes, 1.25 pmole of each forward primer, 5 mM dNTP, 1.5 mM MgCl_2 _and 0.5 U of *Taq *polymerase (Bioman, Taipei, Taiwan). Amplification was conducted as follows: initial denaturation at 95°C for 4 min, followed by 35 cycles at 94°C, 54-58°C and 72°C for 30 sec each. Locus polymorphisms were screened using an ABI PRISM 377 auto DNA sequencer (Applied Biosystems, Foster City, California, USA). Lengths of microsatellite alleles were determined using a TAMRA-labelled 100 bp standard (Perkin-Elmer, Waltham, Massachusetts, USA).

The calculation of the number of alleles, estimated (*H*_E_) and observed heterozygosities (*H*_O_) for each locality and for all samples, the genetic differentiation index (*θ *an estimator of *f *analogues of Wright's fixation indices [43]) among samples, locus-by-locus AMOVA and the exact test for deviations from Hardy-Weinberg equilibrium at every locus for each locality were performed using ARLEQUIN version 3.01 [28] and GENETIX software [44]. The significance level for multiple simultaneous comparisons was adjusted using the sequential Bonferroni technique [45]. The allelic richness of the minimum population size by a rarefaction method and *F*is was estimated with FSTAT 2.9.3 [46]. The significance of the differences in allelic richness was tested by the Wilcoxon signed-rank test for paired observations (see [47] for power analysis). The program GENEPOP version 3.1 [48] was used to test for linkage disequilibrium among the ten loci analysed in this study (10 000 permutations, 1 000 dememorisation steps).

The presence of intraspecific genetic structure was tested using the model-based clustering method [49], as implemented in STRUCTURE VER. 2.1. For each value of K, which is the number of genetically distinct populations, the Markov chain Monte Carlo scheme was run with a burn-in period of 10 000 steps and a chain length of 100 000 replicates following the non-admixture model. Twenty runs were performed to evaluate the reliability of the results, with the number of populations being determined from posterior probabilities of K calculated as K = {1 ~ 6}. The K values could be incorrectly estimated if the migration rates between populations are not equal, so the values of ΔK were also calculated for each value of K [50]. Individuals were regarded as correctly assigned to a population when their q-value (i.e. the posterior probability) was at least 80% after subtracting the posterior probability assignment of another population [47].

## Results

### Mitochondrial DNA

**Figure 2:**
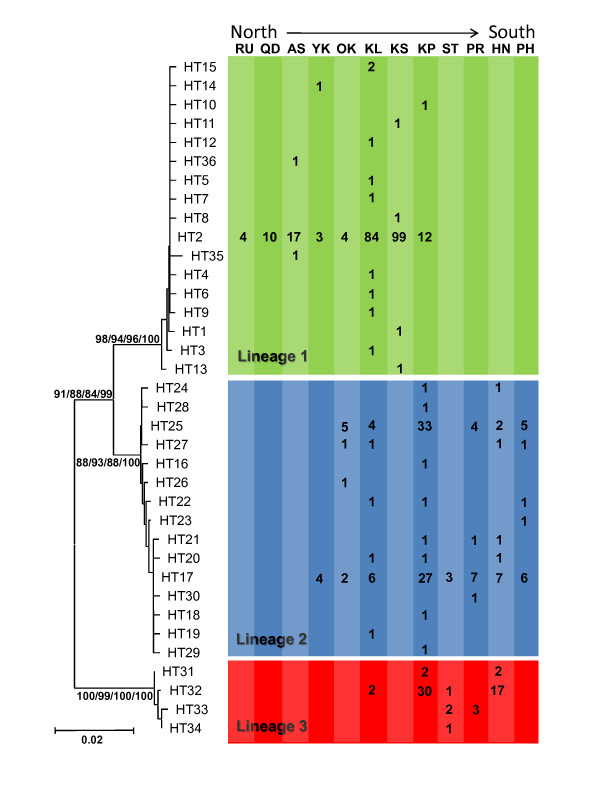
**Phylogenetic trees of *Mugil cephalus *in the northwestern Pacific**. Phylogenetic trees reconstructed from (A) 36 COI sequences haplotypes (HT1-HT36) of *Mugil cephalus *in the northwestern Pacific and its geographic distribution (for location names follow Table 1). The values above the branches are bootstrap values for the NJ, ML and MP analyses and the posterior probabilities for the Bayesian analysis.

**Figure 3:**
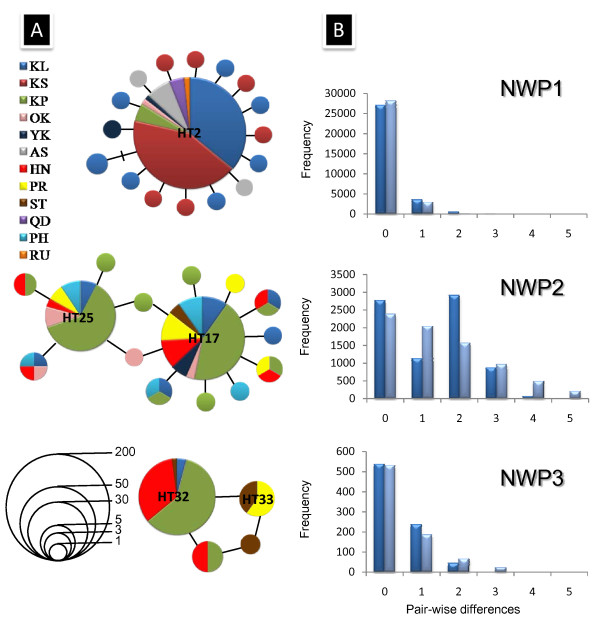
**Haplotype networks and mismatch distribution for three lineages of *Mugil cephalus *COI sequences**. (A) Haplotype networks for three lineages of *Mugil cephalus *COI sequences. The lengths of the connecting lines are in relation to the number of mutations between haplotypes. Each circle represents a haplotype, with the diameter of the circle proportional to the number of sequences of that haplotype. The names of the major haplotypes for each cryptic species correspond to those listed in Table S1. (B) Mismatch distributions from the mtDNA COI sequences of *M. cephalus *from the ten sampling locations. Blue bar: observed distributions; Light blue bar: expected distributions from the sudden expansion model.

A total of 627 nucleotides of the COI gene were obtained from 448 individuals. In these sequences, 54 positions were variable (Additional file 2, Table S1), defining 36 haplotypes (Genbank accession numbers GU260664-GU260697, HQ149082-HQ149083). Phylogenetic trees reconstructed using either COI (Figure 2) or cytochrome *b *(Additional file 3, Figure S2) sequences [17] all supported the existence of three highly supported monophyletic lineages in NW Pacific *M. cephalus*. Seventeen COI haplotypes were found in lineage 1, including 15 nucleotide transitions and three nucleotide transversions. Of these, HT2 was the most frequent haplotype, being shared by the samples from Taiwan, Japan, Qingdao and Russia, and it was connected to the other 16 haplotypes (HT1-HT15, HT35-HT36) by one to two steps in a "star-like" haplotype network (Figure 3a). The other 16 COI haplotypes were minor and mainly found in Keelung and Kaohsiung. Fifteen COI haplotypes were found in lineage 2, including 11 nucleotide transitions and two nucleotide transversions. Two major haplotypes (HT17 and HT25) were shared by samples from Keelung, the Kaoping River, Okinawa, Yokosuka, the Pearl River, Shantou and the Philippines. These two main haplotypes had only two nucleotide differences and were mainly found at the Kaoping River site. Lineage 3 consisted of only four COI haplotypes, with the main haplotype (HT32) occurring at the Kaoping River and Hainan sites and in a few samples from Shantou and Keelung. The fixation index (Φ_ST_) showed significant differentiation among the three lineages (Φ_ST _= 0.973, *P *< 0.001). The pair-wise Φ_ST _values were 0.960 (*P *< 0.001) between lineage 1 and lineage 2; 0.956 (*P *< 0.001) between lineage 2 and lineage 3 and 0.994 (*P *< 0.001) between lineage 1 and lineage 3.

Fifteen variable sites were found within the amplified 627-bp COI fragment (2.5% of sites) between lineage 1 and lineage 2, with approximately 6.7% of the inferred changes being transversions. While 24 and 30 variable sites were found between lineages 2 and 3 (3.8%) and between lineages 1 and 3 (4.8%), respectively, with approximately 21% and 13% of the inferred changes being transversions. The calibrated mean mutation rate of the COI gene in *M. cephalus *was 1.6% mutations/site/MY. Therefore, the calibrated divergence time using BEAST was 1.607 MY (HPD 95% confidence interval were 2.383-0.892 MY, ESS = 1475) between lineages 1 and 2 and 4.200 MY (HPD 95% confidence interval were 5.708-2.801 MY, ESS = 876) between lineage 3 and the ancestor of lineages 1 and 2. BEAST also suggested that lineage 3 is much older than the others, even compared to *M. cephalus *populations present in the eastern Pacific and western Atlantic, and does not share the most recent common ancestor of lineages 1 and 2.

The mismatch distributions of lineage 1 and 3 were unimodal, with almost no pair-wise differences between them (Figure 3b). Lineage 3 exhibited a more uneven mismatch distribution compared to lineage 1. By contrast, the mismatch distribution of lineage 2 was bimodal (Figure 3b), with peaks at zero and two substitutions. RIs were not significant for any lineage. On the other hand, both Tajima's *D *and Fu's *F*_S _were negative and highly significant for lineage 1. For lineage 2, Tajima's *D *values were all negative, but only significant for the control region, while Fu's *F*_S _were all negative and significant for all genes. Finally, both Tajima's *D *and Fu's *F*_S _were negative, but not significant for the lineage 3 for any gene considered (Table 3).

**Table 3:** Summary of the genetic diversity of *Mugil cephalus*.

Species	Marker	n	*N*a/H	*h*/*H*_O _+/- se	π +/- se	RI	Tajima's *D*	Fu's *F*s
NWP1	CR^1^	79	79	1.0000 +/- 0.0020	0.0095 +/- 0.0050	0.008	-2.793***	-24.76***
	Cyto*b*^2^	35	6	0.2689 +/- 0.0982	0.0003 +/- 0.0003	0.285	-2.007**	-5.866***
	COI	250	17	0.1316 +/- 0.0295	0.0002 +/- 0.0004	0.581	-2.439***	-3.4 × 10^38^***
	Microsatellite	390	17.2	0.6097 +/- 0.2751	-	-	-	-

NWP2	CR^1^	47	47	1.0000 +/- 0.0040	0.0364 +/- 0.0180	0.003	-1.962*	-23.263***
	Cyto*b*^2^	43	24	0.9513 +/- 0.0176	0.0030 +/- 0.0017	0.028	-1.68	-16.53***
	COI	138	15	0.6525 +/- 0.0264	0.0021 +/- 0.0015	0.149	-1.169	-7.212**
	Microsatellite	255	13.2	0.5745 +/- 0.2702	-	-	-	-

NWP3	CR^1^	0	-	-	-	-	-	-
	Cyto*b*^2^	17	11	0.9044 +/- 0.0572	0.0052 +/- 0.0030	0.08	-0.783	-1.875
	COI	60	4	0.2989 +/0.0737	0.0006 +/- 0.0006	0.245	-0.361	-1.617
	Microsatellite	68	5.8	0.3310 +/- 0.2991	-	-	-	-

Number of individuals (n), number of haplotype (H) or alleles (*N*a), haplotype diversity (*h *± standard deviation) or observed Heterozygosity (*H*_O _± standard deviation), nucleotide diversity (*π *± standard deviation) for each group of samples. Tajima's *D *and Fu's *F*s, the corresponding P value, and the raggedness index (RI) of these lineages were also indicated.

^1 ^data from [14], ^2 ^data from [17]. *P < 0.05, ** *P *< 0.01, ***P < 0.001

### Microsatellites

A total of 180 alleles with an average of 18.0 alleles per locus were observed across the ten microsatellite loci (Additional file 4, Table S2). All loci were polymorphic, except for locus Mce-8, which was monomorphic in some of the samples. Twenty-one of 180 sample-locus combinations deviated significantly from Hardy-Weinberg proportions after Bonferroni correction. However, the deviations of these loci were not significant in most locations (including Kaoping and Hainan) when individuals were sorted according to one of the three mtDNA lineages, suggesting a Wahlund effect, which is the reduction of heterozygosity in a sample due to the mixture of genetically differentiated populations. No linkage disequilibrium was detected within the *M. cephalus *populations (*P *> 0.05).

AMOVA indicated that the genetic differentiation of *M. cephalus *among 18 spatial and temporal samples was highly significant (*θ *= 0.060, *P *< 0.001). A locus-by-locus AMOVA indicated that all loci could detect population differentiation among the samples. However, no temporal variation was observed in allelic frequencies among the KS, KL and KP locations where the temporal samples were obtained. During three to four years of sampling, no genetic heterogeneity was observed among the temporal samples collected at the same location, except in KP, and the genetic differentiation between locations remained stable (Table 4).

**Table 4:** Temporal pair-wise *θ *test, using microsatellite loci between different sampling locations and years in Taiwan.

			KL			KS			KP		
					
Location	Year	(n)	2005	2006	2007	2005	2006	2007	2005	2007	2008
KL	2005	50	-	0.000	-0.003	0.003	0.004	0.001	0.131***	0.068***	0.061***
	2006	48		-	0.004	-0.001	0.002	0.000	0.150***	0.087***	0.079***
	2007	47			-	0.003	0.007*	0.007**	0.116***	0.055***	0.047***
KS	2005	44				-	0.001	0.000	0.157***	0.090***	0.080***
	2006	50					-	0.001	0.154***	0.088***	0.085***
	2007	57						-	0.165***	0.099***	0.091***
KP	2005	45							-	0.021*	0.041**
	2007	49								-	0.003
	2008	38									-

KL:Keelung; KS:Kaohsiung; KP:Kaoping River.

* < 0.05, ** < 0.01, *** < 0.001 in bold significant values after bonferroni correction [45]

**Figure 4:**
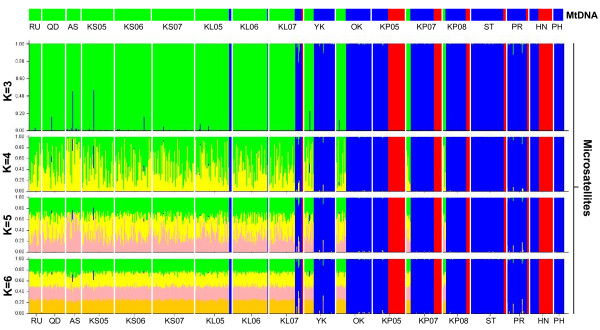
**Correspondence between mtDNA lineages and nuclear Structure clusters**. First row is the distribution of the 3 mtDNA lineages (green: lineage 1, blue: lineage 2, red: lineage 3) in 18 samples of *Mugil cephalus *in NW pacific. Second to forth row are the results of the assignment test using STRUCTURE [49] for *M. cephalus *microsatellite data. Each cluster (K) is designated by a different colour with vertical bars representing individuals and the proportion of a bar assigned to a single colour representing the posterior probability that an individual is assigned to that cluster. Assignment results are shown with K = 3, 4, 5 and 6.

**Figure 5:**
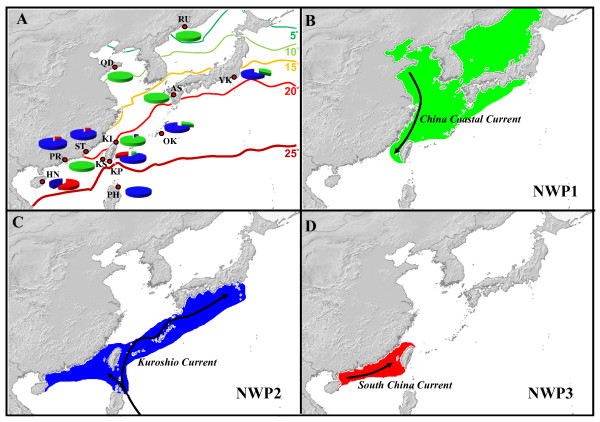
**The distribution of three cryptic species of *Mugil cephalus *in the northwestern Pacific**. (A) the proportions of the three species in various locations; coloured lines indicate sea surface temperature in the NW Pacific in December, which is the period of reproductive migration of *Mugil cephalus*; (B) the distribution range of NWP1 and location of the China Coastal Current; (C) the distribution range of NWP2 and location of the Kuroshio Current; (D) the distribution range of NWP3 and location of the South China Sea Current.

The results from the STRUCTURE assignment test supported three clusters based both on the log probability of the data [L(K)] and the statistic ΔK, as described in [49,50], respectively (Additional file 5, Table S3). With K = 3, almost all individuals were clearly assigned to one of the three clusters (Figure 4). The posterior probabilities corresponding to the assignment of individuals to cluster 3 (red bar, hereafter denoted as NWP3) were all unambiguous, while the assignments to one of the two other clusters (green bar for NWP1 and blue bar for NWP2) included several ambiguous individuals (7 over 645 individuals belonging either to NWP1 and NWP2). The individuals from Kaohsiung, Qingdao, the Ariake Sea and Russia were mostly assigned to NWP1; the individuals from the Philippines were all assigned to NWP2, while at least two clusters coexist in Keelung and the Kaoping River of Taiwan (NWP1, NWP2 and NWP3), Hainan, the Pearl River and Shantou of China (NWP2 and NWP3) and Okinawa and Yokosuka of Japan (NWP1 and NWP2) (Figure 5a). According to their occurrence areas, NWP1 is mainly observed in the north of our sampling area (Figure 5b, North China Seas and Japan), whereas the NWP3 is mostly found at southern stations (Figure 5d, South China Sea). NWP2 has the widest range; it is present from the north to the south of the sampling area (Figure 5c).

### Genetic variability in mtDNA and microsatellites

There was congruence between the two molecular markers when the results of the assignment test were compared to the COI sequence data (Figure 4). The results indicated that 385 of 390 individuals assigned to NWP1 harboured mtDNA representing lineage 1, 253 of 255 individuals assigned to NWP2 harboured mtDNA of lineage 2, and all 68 individuals assigned to NWP3 harboured lineage 3 mtDNA. This resulted in a successful assignment rate of 99.0%. Although there were seven ambiguous individuals in NWP1 and NWP2 below our assignment criteria, the mean q-values of assignment test for these two clusters were still over 0.99 (Additional file 6, Table S4).

In each location sampled, individuals belonging to the same mtDNA lineages were grouped together to further investigate the genetic structure of the three Operational Taxonomic Units (OTUs). Genetic variability analysis showed that only two of the 30 combinations, loci Mce-3 and Mce-8 of NWP3, deviated significantly from Hardy-Weinberg proportions after Bonferroni correction (Additional file 7, Table S5). The allelic richness per locus based on the minimum sample size (68 individuals of NWP3) was significantly lower at NWP3 than NWP2 (*P *< 0.001) and NWP1 (*P *< 0.001) and slightly lower at NWP2 than NWP1 (*P *< 0.05) for the ten microsatellite loci (*H*_O _was lower at NWP3 than the other two clusters). The genotypes at Mce-14 of NWP2 and Mce-10 of NWP3 were all homozygous.

Population genetic differentiation among these three clusters was highly significant (*θ *= 0.179, *P *< 0.001). Pair-wise *θ *comparison indicated that NWP3 was the most differentiated (*θ *_NWP3-NWP1 _= 0.296, *P *< 0.001; *θ *_NWP3-NWP2 _= 0.305, *P *< 0.001), while level of differentiation was lower but still highly significant between NWP1 and NWP2 (*θ *= 0.115, *P *< 0.001). In addition, no genetic heterogeneity was detected among samples belonging to any one of the three OTUs, with the exception of the Ariake Sea, which had some genetic heterogeneity among the samples of NWP1 (Table 5).

**Table 5:** Pair-wise *θ *test, using microsatellite loci between different sampling locations under the same or different mtDNA lineages.

			Lineage 1 (L1)							Lineage 2 (L2)								Lineage 3 (L3)				
					
	Locations	(n)	QD	AS	KL	KS	KP	YK	OK	KL	KP	YK	OK	ST	PR	HN	PH	KL	KP	ST	PR	HN
	RU	17	-0.002	0.008	0.003	0.001	-0.003	-0.001	-0.002	0.096***	0.121***	0.113***	0.099***	0.122***	0.102***	0.130***	0.107***	0.268***	0.390***	0.311***	0.258***	0.279***
	QD	32		0.011*	0.004	0.002	-0.002	-0.011	0.007	0.103***	0.116***	0.117***	0.101***	0.117***	0.102***	0.133***	0.109***	0.271***	0.372***	0.310***	0.261***	0.268***
	AS	21			0.011**	0.016***	0.008	0.000	-0.003	0.086***	0.102***	0.098***	0.087***	0.100***	0.087***	0.112***	0.090***	0.247***	0.367***	0.290***	0.239***	0.255***
L1	KL	129				0.001	-0.006	-0.003	-0.001	0.107***	0.120***	0.121***	0.103***	0.119***	0.100***	0.133***	0.114***	0.261***	0.322***	0.296***	0.246***	0.248***
	KS	151					-0.002	-0.003	0.001	0.112***	0.124***	0.124***	0.107***	0.122***	0.106***	0.138***	0.119***	0.273***	0.330***	0.309***	0.261***	0.257***
	KP	12						-0.010	0.002	0.108***	0.121***	0.125***	0.103***	0.124***	0.101***	0.139***	0.111***	0.270***	0.399***	0.317***	0.258***	0.275***
	YK	14							0.002	0.108***	0.115***	0.117***	0.100***	0.114***	0.100***	0.133***	0.108***	0.269***	0.395***	0.315***	0.258***	0.274***
	OK	14								0.098***	0.125***	0.122***	0.100***	0.123***	0.105***	0.137***	0.109***	0.265***	0.395***	0.313***	0.253***	0.276***
																						
	KL	14									-0.001	-0.002	-0.004	0.004	0.005	0.005	-0.013	0.274***	0.403***	0.324***	0.268***	0.290***
	KP	80										0.000	0.000	-0.002	0.001	0.009	0.002	0.273***	0.342***	0.313***	0.268***	0.261***
	YK	29											0.000	0.000	0.002	-0.002	0.002	0.286***	0.381***	0.329***	0.282***	0.284***
L2	OK	34												-0.002	-0.004	0.000	-0.004	0.264***	0.365***	0.312***	0.263***	0.267***
	ST	44													-0.005	-0.002	0.001	0.280***	0.355***	0.316***	0.269***	0.264***
	PR	27														-0.002	-0.002	0.255***	0.355***	0.299***	0.246***	0.253***
	HN	13															-0.008	0.289***	0.390***	0.327***	0.267***	0.282***
	PH	14																0.251***	0.379***	0.303***	0.242***	0.267***
																						
	KL	2																	0.026	-0.049	-0.095	0.006
	KP	40																		-0.026	-0.026	0.019*
L3	ST	4																			-0.118	-0.003
	PR	3																				-0.017
	HN	19																				

See Table 1 for the complete name of locations.

* < 0.05, ** < 0.01, *** < 0.001 in bold significant values after bonferroni correction [45]

## Discussion

### Historical demography and phylogeography

Since the Plio-Pleistocene, successive periods of sea-level regression have directly impacted the species connectivity of the marginal seas of the NW Pacific. This has clearly been demonstrated by previous phylogeographic studies (Table 1), which have identified Pleistocene refugia in all marginal seas. Our results argue for the existence of three mtDNA lineages of *M. cephalus *in the NW Pacific but only two, NWP1 and NWP2, are of Pleistocene origin. The NWP3 lineage diverged from the NWP1 and NWP2 common ancestor during the Miocene or the Pliocene epoch, and we assume that the origin of NWP3 does not relate to geological events in the NW Pacific because this lineage is also observed in the southwestern (SW) Pacific (in Fiji and New Caledonia Islands; Durand, pers. com.). While it appears likely that the refugium of the NWP2 lineage was located in the South China Sea, the location of the NWP1 refugium is more difficult to assess. The closure of the Tsushima Straits (between Korea and Japan, Figure 1), which isolated the Japan Sea, may have been the powerful vicariant event that was at the origin of the NWP1 lineage. Reconstruction of the history of the Tsushima Current [9] demonstrated that during the interval between 3.5-1.7 MY, the current only flowed into the Sea of Japan periodically and the volume and salinity of water was lower than at present. After 1.7 MY, the Tsushima Current flowed throughout almost all interglacial periods and, more importantly, with a volume and water salinity similar to the present day. This date matches the calibrated dates of divergence estimated between NWP1 and NWP2, suggesting that the NWP1 ancestor was not able to enter in the Japan Sea before 1.7 MY. During each glacial episode, ancestors of the NWP1 lineage that were trapped in the Japan Sea probably experienced several demographic crashes due to decreases in surface water temperatures [51-53]. This hypothesis is consistent with the observed genetic diversity and Tajima's *D *and Fu's *F*_S _indices, which indicate bottleneck events in NWP1 (Table 3). Climatic oscillations during the Pleistocene epoch were probably less marked in more southerly latitudes, where the influence of the Kuroshio Current was still exerted, which would explain why NWP2 presents a higher genetic diversity than NWP1. Similar trends can be observed in the genetic diversity pattern of other NW Pacific species, such as *Chelon haematocheilus *and *Odontamblyopus lacepedii sensu lato*, suggesting that this scenario may have affected all species inhabiting the area [15,16].

Lastly, the East China Sea, which was exposed during the Pleistocene glaciations, seems to be a post-glacial contact zone for *M. cephalus*. While the Yellow Sea was recolonised by NWP1 from a northern route of dispersion through the Tsushima Strait, the Ryukyu Islands and East Japan were recolonised by both NWP1 and NWP2 from southern routes that followed the Kuroshio Current (but see below). If the East China Sea experienced a post-glacial colonisation wave from the Japan Sea, it appears that in species such as *Chelon haematocheilus *and *Eriocheir sensu stricto*, older colonisation waves were also possible, which allowed the emergence of a specific East China Sea lineage in these species [16,54].

### Cryptic *M. cephalus *species in the NW Pacific

It has previously been hypothesised that *M. cephalus *is a species complex rather than a unique panmictic unit. To date, however, this has not been proven, due to inadequate sampling schemes with samples separated by thousands of km, or the use of only maternally inherited markers [14,17,25,31,55-57]. The present study demonstrates that *M. cephalus *is indeed composed of at least three genetically divergent species in the NW Pacific. While COI inter-lineage divergence in *M. cephalus *exceeds intra-lineage divergence (0.4 to 0.2%) by a ratio greater than 10, which is an indicator of cryptic species according to the 10× rule [58], the nuclear bi-parentally inherited markers provide the most definitive evidence of cryptic species. The assignment tests strongly supported the existence of three groups where 99% of *M. cephalus *were significantly assigned (posterior probabilities <10% and >90%). Furthermore, the complete congruence between nuclear groups and mitochondrial lineages suggests that lineage sorting of ancestral polymorphisms has been completed. Lastly, further to the argument that the *M. cephalus *mitochondrial lineages are true species, the gene flow estimated with microsatellite loci appeared to be more limited among sympatric individuals belonging to different mtDNA lineages than between geographically remote individuals sharing the same mtDNA lineage. These results reveal the marked genetic isolation of the three *M. cephalus *species in the NW Pacific.

If, as suggested earlier, the genetic differentiation of these cryptic species is related to past physical barriers to gene flow, the absence of genetic introgression suggests active reproductive isolation where they coexist sympatrically. Previous studies have reported various different reproductive behaviours in *M. cephalus *in the NW Pacific. While some individuals migrate over large geographic scales, others appear to be year-round residents of estuaries [59-64]. Migration was observed over the winter solstice (middle of December-early January), individuals of of 3-4 years of age (FL = 43-55 cm) migrate with the cold North China Coastal Current from the Eastern China Sea to south of the Taiwan Strait to spawn offshore of Kaohsiung, Taiwan [59-61]. The newly hatched larvae drift with the coastal currents and then recruit into the estuaries of the western coast of Taiwan at one to two months post-hatching [59,62-64]. The presence of *M. cephalus *juveniles in Taiwanese estuaries earlier in the year (end of autumn) was interpreted, therefore, as evidence of the existence of a resident *M. cephalus *population [22].

In this study, all individuals sampled from the supposed spawning ground of migrant *M. cephalus *(offshore waters of Kaohsiung) in the spawning season (end of December), over three successive years, belonged to NWP1. The high GSI of these individuals suggested that they were spawning or would spawn soon. By contrast, *M. cephalus *collected in estuarine or coastal waters in the same period belonged to NWP1, NWP2 and NWP3, but all exhibited low GSI (Table 2). This suggests that genetic isolation among *M. cephalus *species is maintained by spatial and temporal differences in spawning migration patterns.

### Population structure and contemporary gene flow

No nuclear genetic heterogeneity was recorded within the cryptic *M. cephalus *species among the locations sampled within the NW Pacific. This findings contrast with those of Liu et al. [23], who documented up to four regional groups of *M. cephalus *along the Chinese coast. However, because we analysed samples from the same locations (Hainan, Shantou and Qingdao, which is a station close to Lianyungang), it is evident that the population structure revealed using AFLP [23] relied mostly on the abundant difference of the three cryptic species. Whereas the Hainan, Shantou and Qingdao samples were characterised by high abundances of NWP3, NWP2 and NWP1, respectively, Liu et al. [23] described them as belonging to 3 populations: IV, II and III, respectively. If there was no experimental artefact, according to Liu et al. [23], a northern population of NWP2 is expected to be isolated in the Bohai Sea (population I), as this population is genetically close to population II.

Finally, if all cryptic *M. cephalus *species revealed in this study are able to disperse and migrate at sea, low genetic structuring is not surprising because large oceanographic current systems in the NW Pacific are expected to greatly facilitate larval dispersal over considerable distances. However, dynamic oceanographic systems can also profoundly restrict connectivity among groups, genetic heterogeneity is generally increased in marine zones with predictable oceanographic fronts [65-67].

The distributional range of the three NW Pacific *M. cephalus *species appears mainly to be shaped by three major oceanographic current systems, namely the South China, North China Coastal and Kuroshio Currents. The range of the NWP2 species appears to match the circulation pattern of the Kuroshio Current, which brings warm tropical water northeastward from the Luzon Straits, past both the west and east coasts of Taiwan and, finally, to Japan, where a branch of the current (the Tsushima Current) enters the Japanese Sea [68,69]. As the world's second-largest ocean current, the Kuroshio has a major effect on other current systems in the area. It, however, mainly remains offshore, whereas the other currents are located on the Taiwanese and North China shelves, and are more coastal. Thus, the South China Current, which flows northward from the South China Sea to the Taiwan Strait, brings warm water which mixes with cold water from the North China Coastal Current flowing southward from the Yellow Sea and East China Sea. The NWP3 distribution range seems to follow the warm water of the South China Current, while the NWP1 species appears to be restricted to the cold waters of the North China Coastal Current, which flows southward (with the northeastern monsoon) during the winter. Such close relationships between oceanographic current systems and fish species distributions presumably reflect temperature preferences, because differences between the Kuroshio Current and the China Coastal Current can range from 6 to 11°C ([70], Figure 5a). While the Kuroshio Current remains warm throughout the year (23-26°C), the China Coastal Current exhibits a wide variation in temperature, from 12°C in March to 20°C in September. In this context, NWP1 may provide a particularly good indicator species for global warming. NWP1 individuals are assumed to spawn at the front (19-21°C) between the cold southward flowing North China Coastal Current and a warm branch of the northward flowing Kuroshio Current, with the front shifting according to the strength of the North China Coastal Current [20,59,60].

## Conclusions

This study successfully identified three cryptic *M. cephalus *species in the NW Pacific Ocean, using both microsatellites and mitochondrial genetic markers. The genetic architecture and current distribution ranges of these species suggest a complex interaction of contemporary and evolutionary factors. Allopatric and adaptive speciation probably occurred during the Pliocene and Pleistocene epochs, when the Sea of Japan was periodically isolated and influenced by cold northern oceanographic currents. After the last glacial maxima, species range expansions were probably facilitated by changes in the oceanographic current structure and the species' temperature preferences. Currently, these species overlap in their distribution ranges, and it is likely that the genetic integrity of each is maintained by temporal and spatial isolation during the spawning period. This study emphasizes the importance of historical separations of marginal seas, in conjunction with fluctuating temperatures, in creating aquatic biodiversity in the NW Pacific. Isolation and demographic fluctuations are expected to constitute a powerful evolutionary force that increases speciation processes in the marine environment. These regional results also shed new light on the potential genetic diversity of *M. cephalus *worldwide.

## Authors' contributions

KNS carried out the molecular genetic studies, performed the statistical analysis and drafted the manuscript. BWJ and CCH participated in the collection of samples and biological data. WNT participated in the design of the study and obtained funding. JDD conceived the study, participated in its design and coordination, and helped to draft the manuscript. All authors have read and approved the final version of the manuscript.

## Supplementary Material

Additional file 1 **Table S1**. Variable positions in the 627 bp mitochondrial COI gene segment of *Mugil cephalus *from 12 locations in the northwestern Pacific. Dots represent identical nucleotides relative to haplotype 1. Frequency of each haplotypes for each lineages (NWP1, NWP2 and NWP3) are also shown. Different color means the locations of the lineage specific nucleotides. Click here for file

Additional file 2 **Table S2**. Genetic variability at ten microsatellite loci of *Mugil cephalus *collected in the northwestern Pacific for spatial and temporal genetic structure test. Table-wide significance levels were applied using the sequential Bonferroni technique [45]. Click here for file

Additional file 3 **Table S3**. Log probability and ΔK [50] for each number of clusters in the Bayesian assignment test as implemented in STRUCTURE [49]. Click here for file

Additional file 4 **Table S4**. The mean q-values and standard deviations (sd) for assignment test of 3 *Mugil cephalus *cryptic species (NWP1, NWP2 and NWP3) as implemented in STRUCTURE [49]. Click here for file

Additional file 5 **Table S5**. Genetic variability at ten microsatellite loci of *Mugil cephalus *among 3 cryptic species. Table-wide significance levels were applied using the sequential Bonferroni technique [45]. Click here for file

Additional file 6 **Figure S1**. Ethidium bromide stained 2% agarose gel showing the multiplex COI haplotype-specific PCR (MHS-PCR) for the rapid screening of three *Mugil cephalus *cryptic species in the NW Pacific. 1-13: unidentified individuals belonging either to lineage NWP1 (2), NWP2 (3, 8, 12, 13) or NWP3 (1, 4-7, 9-11). M: 100-bp DNA ladder. Click here for file

Additional file 7 **Figure S2**. Phylogenetic relationships within *Mugil cephalus *recovered from 1140 bp of the cytochrome *b *sequences according to the neighbour-joining tree using Kimura 2 parameter distance. Leaves of the tree correspond to haplotypes of *M. cephalus *observed by Ke et al. [17] (H1-H43) and this study (H44-H55, in bold). The values above the branches are bootstrap support (500 replicates). Bootstrap supports higher than 50% are displayed. Click here for file
