# Supplementary figures and images for: Plio-Pleistocene sea level and temperature fluctuations in the northwestern Pacific promoted speciation in the globally-distributed flathead mullet Mugil cephalus
Source: BMC Evol Biol. 2011 Mar 31;11:83. doi: 10.1186/1471-2148-11-83 (PMC3079632; doi:10.1186/1471-2148-11-83)

Additional file1, Figure S1

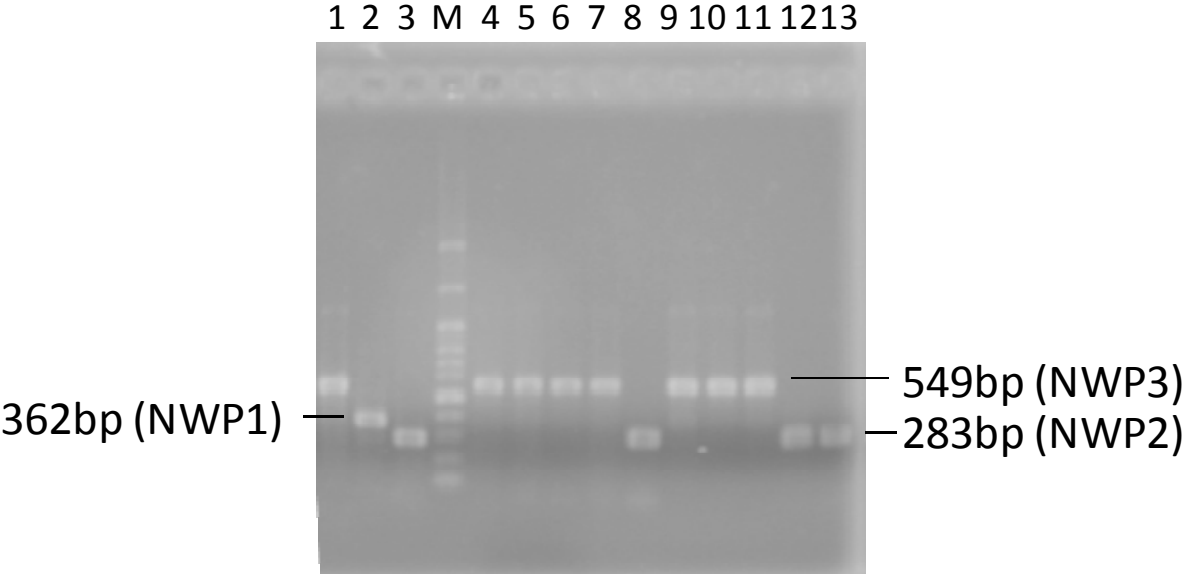

Supplement: Additional file 1 — Table S1. Variable positions in the 627 bp mitochondrial COI gene segment of Mugil cephalus from 12 locations in the northwestern Pacific. Dots represent identical nucleotides relative to haplotype 1. Frequency of each haplotypes for each lineages (NWP1, NWP2 and NWP3) are also shown. Different color means the locations of the lineage specific nucleotides. [file 1471-2148-11-83-S1.PDF]

Additional file 3, Figure S2

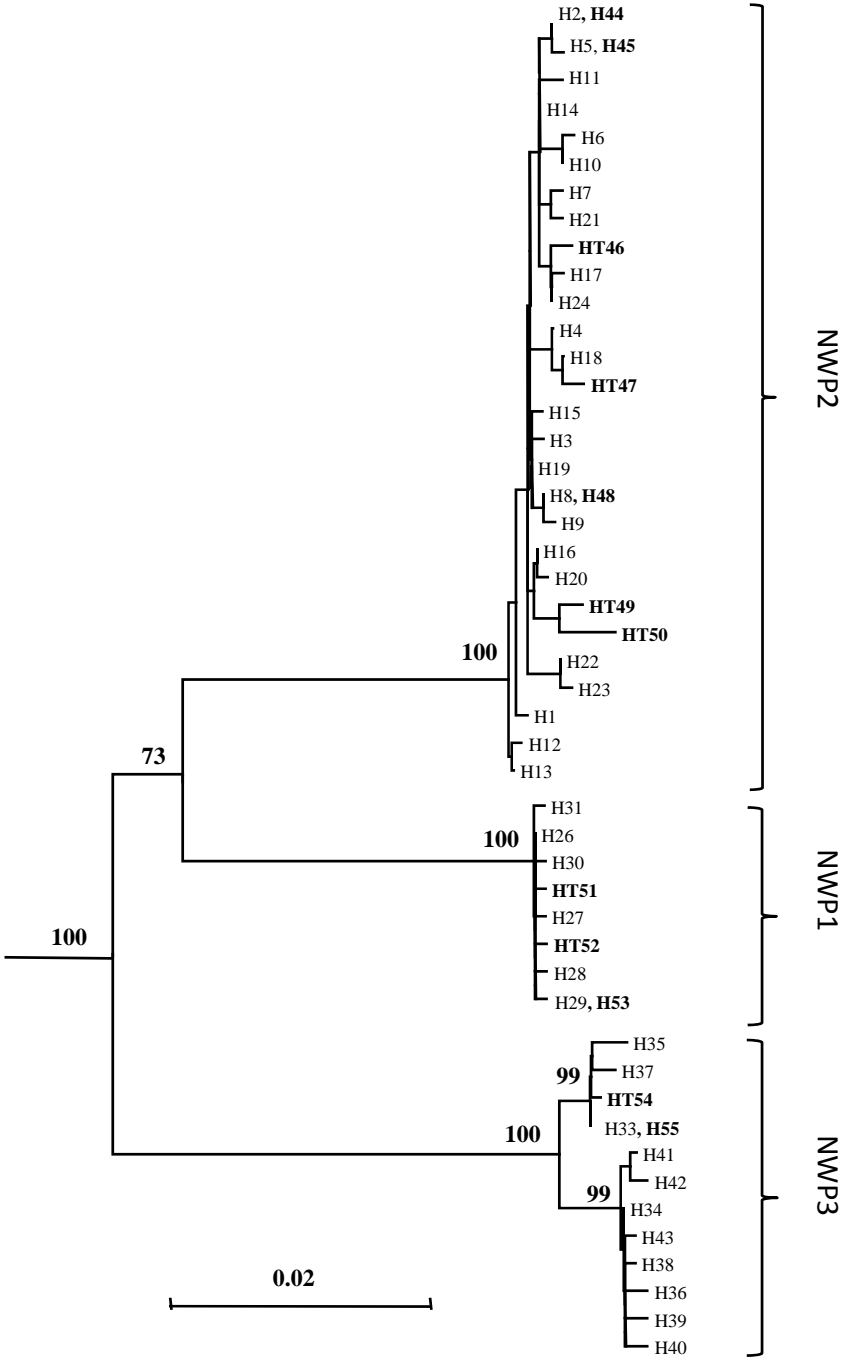

Supplement: Additional file 3 — Table S3. Log probability and ΔK [50] for each number of clusters in the Bayesian assignment test as implemented in STRUCTURE [49]. [file 1471-2148-11-83-S3.PDF]
