# Supplementary material for: Plio-Pleistocene sea level and temperature fluctuations in the northwestern Pacific promoted speciation in the globally-distributed flathead mullet Mugil cephalus
Source: BMC Evol Biol. 2011 Mar 31;11:83. doi: 10.1186/1471-2148-11-83 (PMC3079632; doi:10.1186/1471-2148-11-83)
Supplement: Additional file 2 — Table S2. Genetic variability at ten microsatellite loci of Mugil cephalus collected in the northwestern Pacific for spatial and temporal genetic structure test. Table-wide significance levels were applied using the sequential Bonferroni technique [45]. [file 1471-2148-11-83-S2.PDF]

Additional file 2, Table S1

Table S1 Variable positions in the 627 bp mitochondrial COI gene segment of *Mugil cephalus* from 12 locations in the northwestern Pacific. Dots represent identical nucleotides relative to haplotype 1. Frequency of each haplotypes for each lineages (NWP1, NWP2 and NWP3) are also shown. Different color means the locations of the lineage specific nucleotides.

Haplotypes	NWP	Frequency	Position																																																				
			4	22	59	70	76	85	94	124	139	142	158	161	167	175	205	208	214	232	244	251	256	260	262	265	274	277	280	283	286	301	307	313	314	325	349	352	358	375	389	394	424	427	433	442	454	479	496	502	509	529	535	550	568	577	586
1	1	0.004	T	T	G	G	C	C	A	A	T	G	T	A	C	T	A	T	A	T	T	C	T	T	G	A	T	G	A	A	A	T	A	C	A	C	C	T	C	G	C	C	T	G	T	T	G	C	T	T	T	C	C	T	C	C	G
2	1	0.932	·	·	·	·	·	·	G	·	·	·	·	·	·	·	·	·	·	·	·	·	·	·	·	·	·	·	·	·	·	·	·	·	·	·	·	·	·	·	·	·	·	·	·	·	·	·	·	·	·	·	·	·	·	·	·
3	1	0.004	·	·	·	·	T	·	G	·	·	·	·	·	·	·	·	·	·	·	·	·	·	·	·	·	·	·	·	·	·	·	·	·	·	·	·	·	·	·	·	·	·	·	·	·	·	·	·	·	·	·	·	·	·	·	·
4	1	0.004	·	·	·	·	·	·	G	·	·	·	·	·	·	·	·	·	·	·	·	·	·	·	·	·	·	·	·	·	·	·	·	·	·	·	·	·	·	·	·	·	·	·	·	·	·	·	·	·	·	·	·	C	·	·	·
5	1	0.004	·	·	·	·	·	T	G	·	·	·	·	·	·	·	·	·	·	·	·	·	·	·	·	·	·	·	·	·	·	·	·	·	·	·	·	·	·	·	·	·	·	·	·	·	·	·	·	·	·	·	·	·	·	·	·
6	1	0.004	·	·	·	·	·	·	G	·	·	·	·	·	·	·	·	·	·	·	·	·	·	·	·	·	·	·	·	·	·	·	·	·	·	·	·	·	·	·	·	·	·	·	·	C	·	·	·	·	·	·	·	·	·	·	·
7	1	0.004	·	·	·	·	·	·	G	·	·	·	·	·	·	·	·	·	·	·	·	·	·	·	·	·	·	·	·	·	·	·	·	·	·	·	·	·	T	·	·	·	·	·	·	·	·	·	·	·	·	·	·	·	·	·	·
8	1	0.004	·	·	·	·	·	·	G	·	·	·	·	·	·	·	·	·	·	·	·	·	·	·	·	·	·	·	·	·	·	·	·	·	·	·	T	·	·	·	·	·	·	·	·	·	·	·	·	·	·	·	·	·	·	·	·
9	1	0.004	·	·	·	·	·	·	G	·	·	·	·	·	·	·	·	·	·	·	·	·	C	·	·	·	·	·	·	·	·	·	·	·	·	·	·	·	·	·	·	·	·	·	·	·	·	·	·	·	·	·	·	·	·	·	·
10	1	0.004	·	·	·	·	·	·	G	·	·	·	·	·	·	·	·	·	·	·	·	·	·	·	·	·	·	·	·	·	·	·	·	·	·	·	·	·	·	·	·	·	·	A	·	·	·	·	·	·	·	·	·	·	·	·	·
11	1	0.004	·	·	·	·	·	·	G	·	·	A	·	·	·	·	·	·	·	·	·	·	·	·	·	·	·	·	·	·	·	·	·	·	·	·	·	·	·	·	·	·	·	·	·	·	·	·	·	·	·	·	·	·	·	·	·
12	1	0.004	·	·	·	A	·	·	G	·	·	·	·	·	·	·	·	·	·	·	·	·	·	·	·	·	·	·	·	·	·	·	·	·	·	·	·	·	·	·	·	·	·	·	·	·	·	·	·	·	·	·	·	·	·	·	·
13	1	0.004	·	·	·	·	·	·	G	·	C	·	·	·	·	·	·	·	·	·	·	·	·	·	·	·	·	·	·	·	·	C	·	·	·	·	·	·	·	·	·	·	·	·	·	·	·	·	·	·	·	·	·	·	·	·	·
14	1	0.004	·	·	·	·	·	·	G	·	·	·	·	·	·	·	·	·	·	·	·	·	·	·	·	·	·	·	·	·	C	·	·	·	·	·	·	·	·	·	·	·	·	·	·	·	·	·	·	·	·	·	·	·	·	·	·
15	1	0.008	·	·	·	·	·	·	G	·	·	·	·	·	·	·	·	·	·	·	·	·	·	A	·	·	·	·	·	·	·	·	·	·	·	·	·	·	·	·	·	·	·	·	·	·	·	·	·	·	·	·	·	·	·	·	·
35	1	0.004	·	·	·	·	·	·	G	·	·	·	·	·	·	·	·	·	·	·	·	·	·	·	·	·	·	·	·	·	·	·	C	·	·	·	·	·	·	·	·	·	·	·	·	·	·	T	·	·	·	·	·	·	·	·	·
36	1	0.004	·	·	·	·	·	·	G	·	·	·	·	·	·	·	G	·	·	·	·	·	·	·	·	·	·	·	·	·	·	·	·	·	·	·	·	·	·	·	·	·	·	·	·	·	·	·	·	·	·	·	·	·	·	·	·
16	2	0.007	·	·	·	·	T	·	·	·	C	·	C	G	·	·	·	·	G	·	C	·	·	·	·	·	·	·	·	G	·	·	·	·	·	T	·	C	·	·	T	·	·	·	·	·	A	·	C	·	·	·	A	·	·	·	·
17	2	0.449	·	·	·	·	T	·	·	·	C	·	C	G	·	·	·	·	G	·	C	·	·	·	·	·	·	·	·	G	·	·	·	·	·	T	·	C	·	·	T	·	·	·	·	·	A	·	C	·	·	·	A	·	T	·	·
18	2	0.007	·	·	·	·	T	·	·	·	C	·	C	G	·	·	·	·	G	·	C	·	·	·	·	·	·	·	·	G	·	·	·	·	·	T	·	C	·	·	T	·	·	·	C	·	A	·	C	·	·	·	A	·	T	·	·
19	2	0.007	·	·	·	·	T	·	·	·	C	·	C	G	·	·	·	·	G	·	C	·	·	·	·	G	·	·	·	G	·	·	·	·	·	T	·	C	·	·	T	·	·	·	·	·	A	·	C	·	·	·	A	·	T	·	·
20	2	0.022	·	·	·	·	T	·	·	·	C	·	C	G	·	·	·	·	G	C	C	·	·	·	·	·	·	·	·	G	·	·	·	·	·	T	·	C	·	·	T	·	·	·	·	·	A	·	C	·	·	·	A	·	T	·	·
21	2	0.022	·	·	·	·	T	·	·	·	C	·	C	G	·	·	·	·	G	·	C	·	·	·	·	·	·	·	·	G	·	·	·	·	·	T	·	C	·	·	T	·	·	·	·	·	A	·	C	·	·	·	A	·	T	·	A
22	2	0.022	·	·	·	·	T	·	·	·	C	·	C	G	·	·	·	·	G	·	C	·	·	·	·	·	·	·	·	G	·	·	·	·	·	T	·	·	·	·	T	·	·	·	·	·	A	·	C	·	·	·	A	·	T	·	·
23	2	0.007	·	·	·	·	·	·	·	·	C	·	C	G	·	·	·	·	G	·	C	·	·	·	·	·	·	·	·	G	·	·	·	·	·	T	·	C	·	·	T	·	·	·	·	·	A	·	C	·	·	·	A	·	T	·	·
24	2	0.014	·	·	·	·	T	·	·	·	C	·	C	G	·	·	·	·	G	·	·	·	·	·	·	·	C	·	·	G	·	·	·	·	·	T	·	C	·	·	T	·	·	·	·	·	A	·	C	·	·	·	A	·	·	·	·
25	2	0.384	·	·	·	·	T	·	·	·	C	·	C	G	·	·	·	·	G	·	·	·	·	·	·	·	·	·	·	G	·	·	·	·	·	T	·	C	·	·	T	·	·	·	·	·	A	·	C	·	·	·	A	·	·	·	·
26	2	0.007	·	·	·	·	T	·	·	·	C	·	C	G	·	·	·	·	G	·	·	·	·	·	·	·	·	·	·	G	·	·	·	·	·	T	·	C	·	·	T	·	·	·	·	·	A	·	C	·	·	·	A	·	T	·	·
27	2	0.029	·	·	·	·	T	·	·	·	C	·	C	G	·	·	·	·	G	·	·	·	·	·	·	·	·	·	·	G	·	·	·	·	·	T	·	C	·	·	T	·	·	·	·	·	A	·	C	C	·	·	A	·	·	·	·
28	2	0.007	·	·	C	·	T	·	·	·	C	·	C	G	·	·	·	·	G	·	·	·	·	·	·	·	·	·	·	G	·	·	·	·	·	T	·	C	·	·	T	·	·	·	·	·	A	·	C	·	·	·	A	·	·	·	·
29	2	0.007	·	·	·	·	T	·	·	·	C	·	C	G	·	·	·	·	G	·	C	·	·	·	·	·	·	·	·	G	·	·	·	·	·	T	·	C	·	C	T	·	·	·	·	·	A	·	C	·	·	·	A	·	T	·	·
30	2	0.007	A	·	·	·	T	·	·	·	C	·	C	G	·	·	·	·	G	·	C	·	·	·	·	·	·	·	·	G	·	·	·	·	·	T	·	C	·	·	T	·	·	·	·	·	A	·	C	·	·	·	A	·	T	·	·
31	3	0.067	·	C	·	·	T	·	G	C	C	·	C	G	T	·	·	C	G	C	A	T	·	·	A	·	·	A	G	G	·	·	G	T	G	T	·	C	·	·	T	A	C	·	·	·	·	·	C	·	C	·	·	·	·	T	A
32	3	0.833	·	C	·	·	T	·	G	C	C	·	C	G	T	C	·	C	G	C	A	T	·	·	A	·	·	A	G	G	·	·	G	T	G	T	·	C	·	·	T	A	C	·	·	·	·	·	C	·	C	·	·	·	·	T	A
33	3	0.083	·	C	·	·	T	·	G	C	C	·	C	G	T	C	·	C	G	C	A	T	·	·	A	·	·	A	G	G	·	·	G	T	G	T	·	C	·	·	T	A	C	·	·	·	·	·	C	·	C	A	·	·	·	T	A
34	3	0.017	·	C	·	·	T	·	G	C	C	·	C	G	T	·	·	C	G	C	A	T	·	·	A	·	·	A	G	G	·	·	G	T	G	T	·	C	·	·	T	A	C	·	·	·	·	·	C	·	C	A	·	·	·	T	A
