# Supplementary material for: Plio-Pleistocene sea level and temperature fluctuations in the northwestern Pacific promoted speciation in the globally-distributed flathead mullet Mugil cephalus
Source: BMC Evol Biol. 2011 Mar 31;11:83. doi: 10.1186/1471-2148-11-83 (PMC3079632; doi:10.1186/1471-2148-11-83)
Supplement: Additional file 4 — Table S4. The mean q-values and standard deviations (sd) for assignment test of 3 Mugil cephalus cryptic species (NWP1, NWP2 and NWP3) as implemented in STRUCTURE [49]. [file 1471-2148-11-83-S4.PDF]

Additional file 4, Table S2

Table S2 Genetic variability at ten microsatellite loci of *Mugil cephalus* collected in the northwestern Pacific for spatial and temporal genetic structure test. Table-wide significance levels were applied using the sequential Bonferroni technique [45].

Microsatellite Locus		Keelung 05 (n=50)	Keelung 06 (n=48)	Keelung 07 (n=47)	Kaohsiung 05 (n=44)	Kaohsiung 06 (n=50)	Kaohsiung 07 (n=57)	Kaoping River 05 (n=45)	Kaoping River 07 (n=49)	Kaoping River 08 (n=38)	Okinawa 05 (n=48)	Yokosuka 05 (n=43)	Ariake Sea 07 (n=21)	Hainan 10 (n=32)	Pearl River 05 (n=30)	Shantou 08 (n=48)	Qingdao 05 (n=32)	Philippines 07 (n=14)	Russia 07, 09 (n=17)	Total n=713
Mce-2	No. of alleles	36	35	35	31	32	35	29	31	29	33	30	20	19	26	33	24	17	19	45
	Allele range	101-193	101-177	101-191	101-181	103-179	101-175	105-169	107-177	103-185	105-183	101-175	103-177	111-165	105-181	105-179	105-175	101-171	101-175	101-193
	H_O	0.960	1.000	0.979	0.886	1.000	0.930	0.933	0.980	0.895	0.958	0.930	0.857	0.875	0.867	0.917	1.000	0.857	0.941	0.940
	H_E	0.973	0.966	0.973	0.963	0.960	0.962	0.946	0.967	0.955	0.971	0.961	0.948	0.905	0.964	0.955	0.953	0.968	0.943	0.964
	H.-W. test	n.s.	n.s.	n.s.	n.s.	n.s.	n.s.	n.s.	n.s.	n.s.	n.s.	n.s.	n.s.	n.s.	n.s.	n.s.	n.s.	n.s.	n.s.	*
Mce-3	No. of alleles	24	20	25	20	26	26	13	17	19	19	21	18	12	16	17	18	10	13	36
	Allele range	127-191	127-179	127-195	137-205	127-211	125-187	139-179	135-185	135-205	127-191	135-191	133-195	127-179	127-177	129-187	135-177	127-165	139-177	125-211
	H_O	0.940	0.813	0.915	0.864	0.940	0.860	0.578	0.714	0.737	0.750	0.837	0.857	0.563	0.767	0.729	0.906	0.786	0.824	0.802
	H_E	0.954	0.938	0.951	0.944	0.955	0.942	0.831	0.855	0.872	0.839	0.813	0.930	0.789	0.825	0.797	0.935	0.788	0.934	0.916
	H.-W. test	n.s.	n.s.	n.s.	n.s.	n.s.	n.s.	***	***	n.s.	n.s.	n.s.	n.s.	***	n.s.	n.s.	n.s.	n.s.	n.s.	***
Mce-4	No. of alleles	8	10	8	10	9	11	8	10	11	13	11	10	9	9	11	10	7	6	16
	Allele range	185-201	185-209	181-197	183-207	185-209	183-203	181-207	181-205	181-201	183-217	183-207	181-205	181-201	181-203	181-207	185-207	183-199	185-197	181-217
	H_O	0.580	0.583	0.596	0.477	0.560	0.456	0.311	0.490	0.763	0.750	0.674	0.667	0.313	0.667	0.750	0.781	0.714	0.706	0.588
	H_E	0.680	0.619	0.684	0.696	0.594	0.499	0.708	0.792	0.885	0.818	0.808	0.834	0.672	0.806	0.842	0.757	0.820	0.663	0.795
	H.-W. test	n.s.	n.s.	n.s.	n.s.	n.s.	n.s.	***	***	n.s.	n.s.	n.s.	n.s.	***	n.s.	n.s.	n.s.	n.s.	n.s.	***
Mce-6	No. of alleles	9	9	9	11	9	9	9	9	9	12	10	7	8	7	9	7	6	7	15
	Allele range	187-203	179-201	179-203	179-201	183-203	181-201	189-209	189-209	179-203	181-209	187-205	187-201	187-205	189-203	189-209	189-201	191-203	189-201	179-209
	H_O	0.680	0.750	0.766	0.818	0.820	0.825	0.600	0.653	0.632	0.729	0.814	0.810	0.531	0.767	0.729	0.750	0.357	0.824	0.727
	H_E	0.816	0.826	0.824	0.822	0.804	0.799	0.676	0.768	0.815	0.842	0.821	0.762	0.589	0.779	0.732	0.793	0.759	0.841	0.807
	H.-W. test	n.s.	n.s.	n.s.	n.s.	n.s.	n.s.	***	n.s.	n.s.	n.s.	n.s.	n.s.	n.s.	n.s.	n.s.	n.s.	**	n.s.	***
Mce-7	No. of alleles	20	16	16	16	16	15	12	16	14	17	15	15	14	12	15	14	11	11	26
	Allele range	176-226	184-226	184-224	184-224	180-214	180-220	176-208	176-222	176-210	184-216	176-214	178-212	176-210	176-210	176-210	184-212	176-210	180-218	176-226
	H_O	0.900	0.833	0.787	0.750	0.820	0.772	0.822	0.796	0.711	0.813	0.884	0.905	0.844	0.667	0.729	0.844	0.929	0.882	0.808
	H_E	0.899	0.879	0.900	0.872	0.901	0.873	0.884	0.886	0.844	0.895	0.853	0.906	0.894	0.885	0.875	0.883	0.892	0.891	0.895
	H.-W. test	n.s.	n.s.	n.s.	n.s.	n.s.	n.s.	n.s.	n.s.	n.s.	n.s.	n.s.	n.s.	***	***	n.s.	n.s.	n.s.	n.s.	***
Mce-8	No. of alleles	6	4	4	4	5	5	4	5	4	5	4	4	3	4	3	4	2	4	9
	Allele range	178-188	178-184	178-184	178-188	172-184	172-184	178-184	178-188	178-192	174-184	172-182	172-182	178-182	174-182	178-182	178-184	180-182	178-184	172-192
	H_O	0.360	0.250	0.489	0.386	0.540	0.421	0.111	0.327	0.289	0.354	0.488	0.524	0.313	0.400	0.417	0.406	0.429	0.588	0.383
	H_E	0.448	0.321	0.500	0.352	0.481	0.436	0.572	0.640	0.557	0.424	0.467	0.460	0.601	0.514	0.482	0.383	0.476	0.528	0.509
	H.-W. test	n.s.	n.s.	n.s.	n.s.	n.s.	n.s.	***	***	***	n.s.	n.s.	n.s.	***	n.s.	*	n.s.	n.s.	n.s.	***
Mce-10	No. of alleles	7	8	8	8	5	9	3	6	4	6	7	6	4	4	4	5	4	4	12
	Allele range	134-148	128-148	128-148	134-150	136-144	124-148	136-140	130-144	134-140	134-148	128-146	130-146	134-140	134-140	134-140	136-148	134-140	136-142	124-150
	H_O	0.540	0.500	0.617	0.591	0.480	0.614	0.178	0.408	0.526	0.604	0.349	0.714	0.156	0.200	0.396	0.375	0.643	0.647	0.468
	H_E	0.632	0.568	0.648	0.584	0.477	0.601	0.205	0.403	0.534	0.580	0.511	0.632	0.205	0.301	0.419	0.504	0.563	0.608	0.516
	H.-W. test	n.s.	n.s.	n.s.	n.s.	n.s.	n.s.	n.s.	n.s.	n.s.	n.s.	n.s.	n.s.	n.s.	n.s.	n.s.	n.s.	n.s.	n.s.	n.s.
Mce-11	No. of alleles	6	5	5	6	5	6	4	5	5	5	5	5	3	6	4	6	4	5	7
	Allele range	157-171	161-171	161-171	161-171	161-171	161-171	165-171	161-171	161-171	161-171	161-171	161-171	165-169	161-171	165-171	161-171	161-169	161-171	157-171
	H_O	0.560	0.458	0.596	0.500	0.640	0.614	0.289	0.429	0.526	0.521	0.535	0.571	0.438	0.467	0.354	0.750	0.643	0.529	0.516
	H_E	0.603	0.562	0.634	0.534	0.614	0.570	0.310	0.461	0.474	0.502	0.469	0.635	0.389	0.451	0.358	0.702	0.513	0.610	0.524
	H.-W. test	n.s.	n.s.	n.s.	n.s.	n.s.	n.s.	n.s.	n.s.	n.s.	n.s.	n.s.	n.s.	n.s.	n.s.	n.s.	n.s.	n.s.	n.s.	n.s.
Mce-14	No. of alleles	3	2	1	1	3	2	1	1	1	1	1	2	2	1	1	3	1	2	4
	Allele range	155-161	159-161	161	161	155-161	159-161	161	161	161	161	161	159-161	159-161	161	161	153-161	161	159-161	153-161
	H_O	0.040	0.021	–	–	0.060	0.018	–	–	–	–	–	0.048	0.031	–	–	0.094	–	0.059	0.018
	H_E	0.040	0.021	–	–	0.059	0.018	–	–	–	–	–	0.048	0.031	–	–	0.092	–	0.059	0.018
	H.-W. test	n.s.	n.s.	–	–	n.s.	n.s.	–	–	–	–	–	n.s.	n.s.	–	–	n.s.	–	n.s.	n.s.
Mce-22	No. of alleles	5	4	5	4	4	5	8	7	6	6	6	4	6	7	6	3	5	3	10
	Allele range	116-126	116-122	116-126	116-126	116-122	114-122	110-126	116-128	116-126	116-126	116-126	114-120	112-126	112-126	116-126	116-120	116-126	118-122	110-128
	H_O	0.360	0.458	0.404	0.477	0.380	0.386	0.511	0.510	0.447	0.542	0.465	0.571	0.500	0.567	0.458	0.375	0.429	0.471	0.456
	H_E	0.550	0.471	0.635	0.419	0.421	0.442	0.698	0.728	0.679	0.683	0.673	0.556	0.696	0.594	0.499	0.388	0.484	0.444	0.739
	H.-W. test	***	n.s.	***	n.s.	n.s.	n.s.	***	***	***	***	n.s.	n.s.	***	n.s.	n.s.	n.s.	n.s.	n.s.	***

$*P < 0.05$, $**P < 0.01$, $***P < 0.001$
