# Supplementary material for: Plio-Pleistocene sea level and temperature fluctuations in the northwestern Pacific promoted speciation in the globally-distributed flathead mullet Mugil cephalus
Source: BMC Evol Biol. 2011 Mar 31;11:83. doi: 10.1186/1471-2148-11-83 (PMC3079632; doi:10.1186/1471-2148-11-83)
Supplement: Additional file 5 — Table S5. Genetic variability at ten microsatellite loci of Mugil cephalus among 3 cryptic species. Table-wide significance levels were applied using the sequential Bonferroni technique [45]. [file 1471-2148-11-83-S5.PDF]

Additional file 5, Table S3

Table S3 Log probability and ΔK [50] for each number of clusters in the Bayesian assignment test as implemented in STRUCTURE [49].

K (Number of clusters)	L(K)(Average of log probsbility)	Standard deviation	L'(K)	| L''(K)|	ΔK
1	-25175.02	0.74			
2	-23549.67	93.79	1625.35	182.85	1.95
3	-22107.17	39.27	1442.50	1544.67	39.33
4	-22209.34	45.41	-102.17	31.76	0.70
5	-22343.27	131.84	-133.93	203.84	1.55
6	-22681.04	245.07	-337.77	-337.77	-1.38
