# Supplementary material for: Plio-Pleistocene sea level and temperature fluctuations in the northwestern Pacific promoted speciation in the globally-distributed flathead mullet Mugil cephalus
Source: BMC Evol Biol. 2011 Mar 31;11:83. doi: 10.1186/1471-2148-11-83 (PMC3079632; doi:10.1186/1471-2148-11-83)
Supplement: Additional file 6 — Figure S1. Ethidium bromide stained 2% agarose gel showing the multiplex COI haplotype-specific PCR (MHS-PCR) for the rapid screening of three Mugil cephalus cryptic species in the NW Pacific. 1-13: unidentified individuals belonging either to lineage NWP1 (2), NWP2 (3, 8, 12, 13) or NWP3 (1, 4-7, 9-11). M: 100-bp DNA ladder. [file 1471-2148-11-83-S6.PDF]

Additional file 6, Table S4

Table S4 The mean q-values and standard deviations (sd) for assignment test of 3 *Mugil cephalus* cryptic species (NWP1, NWP2 and NWP3) as implemented in STRUCTURE [49].

Species	mean q-value	sd
NWP1	0.9922	0.0458
NWP2	0.9966	0.0195
NWP3	1	0
