# Supplementary material for: Plio-Pleistocene sea level and temperature fluctuations in the northwestern Pacific promoted speciation in the globally-distributed flathead mullet Mugil cephalus
Source: BMC Evol Biol. 2011 Mar 31;11:83. doi: 10.1186/1471-2148-11-83 (PMC3079632; doi:10.1186/1471-2148-11-83)
Supplement: Additional file 7 — Figure S2. Phylogenetic relationships within Mugil cephalus recovered from 1140 bp of the cytochrome b sequences according to the neighbour-joining tree using Kimura 2 parameter distance. Leaves of the tree correspond to haplotypes of M. cephalus observed by Ke et al. [17] (H1-H43) and this study (H44-H55, in bold). The values above the branches are bootstrap support (500 replicates). Bootstrap supports higher than 50% are displayed. [file 1471-2148-11-83-S7.PDF]

Additional file 7, Table S5

Table S5 Genetic variability at ten microsatellite loci of *Mugil cephalus* among 3 cryptic species. Table-wide significance levels were applied using the sequential Bonferroni technique [45].

Microsatellite Locus		NWP1 n=390	NWP2 n=255	NWP3 n=68
Mce-2	No. of alleles/Allelic richness	45/36.6	42/35.2	17/17
	Allele range	101-193	101-191	109-155
	H_O	0.951	0.953	0.824
	H_E	0.964	0.963	0.874
	H.-W. test	n.s.	n.s.	n.s.
	F_{is}	0.013	0.010	0.058
Mce-3	No. of alleles/Allelic richness	35/26.1	21/16.1	9/9
	Allele range	125-211	127-177	143-187
	H_O	0.900	0.761	0.397
	H_E	0.947	0.744	0.620
	H.-W. test	n.s.	n.s.	***
	F_{is}	0.049	-0.022	0.362
Mce-4	No. of alleles/Allelic richness	14/11.2	15/11.8	4/4
	Allele range	183-209	181-217	181-191
	H_O	0.595	0.706	0.103
	H_E	0.652	0.795	0.113
	H.-W. test	n.s.	n.s.	n.s.
	F_{is}	0.088	0.112	0.093
Mce-6	No. of alleles/Allelic richness	13/10.1	10/9.3	8/8
	Allele range	179-203	189-209	187-205
	H_O	0.774	0.737	0.412
	H_E	0.812	0.791	0.395
	H.-W. test	n.s.	n.s.	n.s.
	F_{is}	0.047	0.068	-0.043
Mce-7	No. of alleles/Allelic richness	26/17.9	17/14.2	7/7
	Allele range	176-226	176-222	176-192
	H_O	0.833	0.776	0.779
	H_E	0.889	0.841	0.791
	H.-W. test	n.s.	n.s.	n.s.
	F_{is}	0.062	0.076	0.015
Mce-8	No. of alleles/Allelic richness	9/5.8	5/3.9	2/2
	Allele range	172-192	174-184	178-180
	H_O	0.428	0.404	0.044
	H_E	0.424	0.430	0.150
	H.-W. test	n.s.	n.s.	***
	F_{is}	-0.009	0.062	0.707
Mce-10	No. of alleles/Allelic richness	12/8.3	8/5.1	1/1
	Allele range	124-150	130-146	136
	H_O	0.554	0.463	0.000
	H_E	0.581	0.491	0.000
	H.-W. test	n.s.	n.s.	—
	F_{is}	0.047	0.058	—
Mce-11	No. of alleles/Allelic richness	7/5.8	6/5.1	3/3
	Allele range	157-171	161-171	165-169
	H_O	0.600	0.439	0.324
	H_E	0.605	0.424	0.309
	H.-W. test	n.s.	n.s.	n.s.
	F_{is}	0.009	-0.036	-0.046
Mce-14	No. of alleles/Allelic richness	4/2.3	1/1	2/2
	Allele range	153-161	161	159-161
	H_O	0.031	0.000	0.015
	H_E	0.030	0.000	0.015
	H.-W. test	n.s.	—	n.s.
	F_{is}	-0.011	—	0.000
Mce-22	No. of alleles/Allelic richness	6/4.4	7/6.2	5/5
	Allele range	114-126	116-128	110-126
	H_O	0.431	0.506	0.412
	H_E	0.444	0.461	0.356
	H.-W. test	n.s.	n.s.	n.s.
	F_{is}	0.031	-0.097	-0.159

***$P < 0.001$
